# Gastrointestinal Helminth Parasites of Chicken under Different Management System in Mekelle Town, Tigray Region, Ethiopia

**DOI:** 10.1155/2019/1307582

**Published:** 2019-02-11

**Authors:** Mebrahtu Berhe, Berhanu Mekibib, Abrha Bsrat, Gebretsadik Atsbaha

**Affiliations:** ^1^Veterinary Drug and Feed Administration and Control Authority, P.O. Box 1776, Mekelle, Ethiopia; ^2^Faculty of Veterinary Medicine, Hawassa University, P.O. Box 05, Hawassa, Ethiopia; ^3^College of Veterinary Medicine, Mekelle University, P.O. Box 2084, Mekelle, Ethiopia

## Abstract

The poultry industry is an infant but fast growing sector in Ethiopia. However, it is largely dependent on local chicken managed under backyard production system. The sector is facing different challenges, mainly emanated from prevalence of infectious diseases such as helminth parasite species. Hence, this study came up with an aim to determine the infection rate and identify helminth parasite species in chickens managed under different production systems, in Mekelle, Ethiopia. A cross-sectional study design was employed, from November 2015 to March 2016. Postmortem (N=138) and fecal (N=410) samples of chicken were considered for necropsy and coproscopic examination to see both adult and eggs of helminth parasites, respectively. Similar gastrointestinal helminth parasites infection rate of chicken was obtained from both examination approaches (necropsy, 90.60%; and coproscopy, 90.97%). The study attested high prevalence (87.7%) of mixed infection with helminth parasites of chicken.* Heterakis gallinarum *(72.5%) and* Ascaridia galli* (68.8%) were found as the most dominant species (necropsy). During coproscopic examination cestode (89%) infections showed a relatively higher prevalence than nematodes (84.4%), although no difference was observed during that of necropsy examination results. Chickens of local breed from backyard production system had shown more likelihood of getting helminth infection when compared with their corresponding relatives (coproscopy). However, the variation was not statistically significant during that of necropsy finding. Therefore, the higher prevalence of parasitism and mixed infection observed in the study area would warrant for an urgent intervention with regular deworming scheme, and strict attention should be given towards hygienic measures and other health related management activities.

## 1. Introduction

Chickens are widely spread at almost every family, in Ethiopia, providing valuable source of protein and cash income. About 99 % of Ethiopian poultry resources managed under backyard production system undergo poor handling scheme. Backyard production system involves low productivity with less input and periodic flock devastation due to different reasons. Women, in the rural are mainly involved in the system to benefit an immediate cash income [1, 2].

Poultry industry in Ethiopia is infant but fast growing sector. The industry faces various challenges such as shortage of feed in terms of quality and quality, poor husbandry practices, prevalence, and wide distribution of infectious and noninfectious diseases. Poor veterinary services and lack of appropriate breeding practices are assumed to be additional challenges. Moreover, the government has given less attention [3, 4]. Disease is resulting in as high as 20% to 50% estimated mortality rate ranges [5].

Parasites are among the infectious agents that cause an alarming problem to the industry, posing adverse economic effects. Gastrointestinal parasitism leads to significant economic losses in poultry [6]. Nematodes cause more serious problem in backyard flocks, in developing countries like Ethiopia. The backyard scavenging production system exposes chickens to certain eggs and larvae of parasites from ingested soils and insects [3, 7]. Helminth infections in rural free-range chicken are ubiquitous and may result in subclinical diseases even when they occur in lower numbers [8].

Recently, poultry farms in Ethiopia are dramatically increasing in number. However, research findings conducted in different parts of the country, incriminated helminthes as major causes of ill health and loss of productivity in local chickens [9]. Meanwhile, there is a definite paucity of information on gastrointestinal parasitic infections of chickens in Mekelle city, Ethiopia. Hence, in order to design effective preventive and control strategies, it is very much essential to know about the available helminth parasite species and their burden on chickens in the study area. Therefore, this study was conducted under intensive, semi-intensive, and backyard management systems, to determine the prevalence of gastrointestinal helminth parasites and identify the parasite species that infect chicken in the study area.

## 2. Materials and Methods

### 2.1. Description of the Study Area

The study was carried out in Mekelle, the capital city of Tigray National Region State, geographically situated between 13°09′′ and 14°34′′ North latitude and 39°12′′ and 40°28′′ East longitude. The area is located some 784 Km away from Addis Ababa in north direction. Agroclimatic condition of the area is midland locally called Weynadega with an elevation of 2254 meters above sea level. The average annual temperature ranges between 11.11°C and 24.1°C. The average annual rainfall is measured to be 570 mm [10].

### 2.2. Study Animal

Chickens under intensive, semi-intensive, and backyard production systems, in Mekelle town, were considered during the study. The studied animals were systematically categorized as per the Ethiopian poultry production systems context [2]. A total number of (N = 410) chickens and (N = 138) chickens were examined for coproscopy and necropsy, respectively. Fecal samples were taken from backyard chickens where their number (n=155) is dominated by local breed that were randomly selected from different households of selected residential sites locally called kebeles. Minimal numbers of the back yard animals under study (n=4) were exotic. Chicken from three semi-intensive production system (n=130) that were from both exotic (n=84) and local breeds (n=46) were kept by smallholders. On the other hand, large number of exotic (Rhode Island Red) chicken breeds (n=125) from two intensive farms were also involved. As to the sex of study animals number is concerned; a total number of 209 and 201 chickens for male and female, respectively, were randomly involved from all forms of the management systems. A considerable number of chickens (N=138) all from the selected kebeles were randomly taken from volunteer restaurants and households with both intensive and backyard management for postmortem examination. As continuation procedure of the postmortem step, gastrointestinal samples were collected at the same span of time used for fecal examination. Among the 138 sample animals, 96 of which were male from local breed that are managed under backyard system and the other 42 ones were female from exotic breed that are managed under intensive system. The higher number of male chicken may be due to the abundant access of male chickens slaughtered during the holidays held at the study period.

### 2.3. Study Design and Sampling Technique

Cross-sectional type of study design was employed from November 2015 to March 2016, with aims to estimate the prevalence of gastrointestinal helminth parasites of chicken and to identify associated risk factors. Postmortem and fecal samples were collected from chickens of all (intensive, semi-intensive, and backyard) production systems. Based on the poultry population potential, Semen and Hawelti (two subcities with 11 kebeles) and other three kebeles from each subcity were purposely selected. Two poultry farms with chickens (n=125) from intensive, three farms with chickens (n=130) from semi-intensive, and other chickens (n=155) from different randomly selected households at selected kebeles with backyard production systems were involved. Fecal samples were collected from (N = 410) chickens out of randomly selected poultry farms. Following this, samples have proportionally allocated to each of the management systems, examined in every after one month of the study period. Furthermore, necropsy examination was conducted on chickens (N = 138) of three restaurants originated from intensive farms (n=42) and from chickens slaughtered (n=96) in randomly selected households during the Ethiopian (christmas and epiphany) holidays. Sex, breed, and type of production systems of each sampled chicken were recorded on a format prepared for this purpose.

### 2.4. Sample Collection and Diagnosis

#### 2.4.1. Coproscopy Examination

Fecal samples were collected directly from the cloaca and the ground immediately after defecation and then placed in a clean universal bottle. Collected samples were preserved with 10% formalin and transported to Mekelle University, College of Veterinary Medicine, Department of Parasitology, for laboratory examination. Flotation technique was involved with NaCl solution that uses the fluid for flotation. Simultaneously, sedimentation technique was employed using a centrifugation (1500 rpm for 3 minutes) [7]. Speciation of the gastrointestinal helminth parasites was done according to the helminthological keys of Soulsby [11].

#### 2.4.2. Necropsy Examination

The whole gastrointestines of chicken were collected from volunteer restaurants and households (during holydays) within the selected kebeles of Mekelle town. The gut samples were collected soon after evisceration and immersed into sample box filled with 10% buffered formalin and transported to the Mekelle University, College of Veterinary Medicine, Parasitology Laboratory Department, for necropsy examination with required information regarding their source. The intestine was opened longitudinally with a scissor and the content for each intestine were carefully scraped into a petri-dish and a small amount of tap water was added to soften whatever debris to facilitate recovery of worms from the lumen. All worms visible to naked eye were removed using a thumb forceps. The recovered worms were put in another petri-dish labeled according to predilection site and 10% ethanol added to help straightening before identification under stereomicroscope using morphological keys described according to [7, 11].

### 2.5. Data Analysis

The data obtained from postmortem and fecal examinations has entered into Microsoft Excel spreadsheet; raw data were coded and then analyzed using STATA [12]. Descriptive analysis was used to determine frequency and percentage of the parasite infections. Logistic regression analysis has been used to estimate and measure the association among possible risk factors and the infection rate of helminth parasites recorded from fecal samples. In all cases P<0.05 was considered statistically significant, at 95% confidence interval. P value was calculated to observe diagnostic potential difference among the parasites species identified by both postmortem and fecal examinations approaches, considering only the samples that originated from the same chicken.

### 2.6. Ethical Consideration

Ethical clearance was obtained from Institutional Review Board of Mekelle University, College of Veterinary Medicine. Furthermore, verbal informed consent was obtained from all poultry farm owners or managers participated in the study, after explaining the purpose of the study in their local language (Tigrigna).

## 3. Result

### 3.1. Coproscopic Examination

Among the 410 chickens examined, 373 (90.98%) were positive for eggs of one or more helminth parasites. Management system and breed factors were significantly associated with an overall infection rate of helminth parasites. However, no significant difference was observed for sex factor (Table 1). When compared with intensively managed one, chicken managed at backyard and semi-intensive management system are four and two times more at risk for helminth parasites infection, respectively. Moreover, local breed chickens are almost 3 times more at risk of getting helminth parasites than their counterparts are. However, the variation observed was not statistically significant, when the collective effects of these variables are adjusted together.

During coproscopy examination, eight (8) helminth parasite species (three nematodes and five cestodes) were identified (Table 2). Those are* Ascaridia galli*,* Heterakis gallinarum, *and* Capillaria* (from nematodes) and* Raillietina tetragona, Raillietina echinobothrida, Raillietina cesticillus, Davainea proglottina, *and* Hymenolepis carioca* (from cestodes). In general, infection rate of the parasite species has shown a relative decrease in backyard, semi-intensive, and intensive management system, respectively. Besides, majority of the helminth parasites encountered was found relatively higher in local breed and male chicken than their counterparts.

Among the 410 chickens examined, infection rate of 89% (365/410) for cestode and 84.4% (346/410) for nematode parasite species were recorded. Management and breed factors were found significantly associated with both cestode and nematode parasitism, though the variation among breeds was not significant when adjusted with the other variables together. Chickens from backyard management system (AOR=4.9, 95%CI=1.1-20.9) and local breed (AOR=1.2, 95%CI= 0.4-3.9) had more chance to get cestode infection and, similarly, these chickens had also (AOR=3.8, 95%CI=1.1-12.9 and AOR=1.1, 95%CI=0.4-3.1) more likelihood of getting nematode infections compared with their counterparts, respectively (Table 3).

### 3.2. Necropsy Examination

Out of 138 chickens' gut dissected for adult helminth parasites examination, 125(90.6%) were found harboring one or more species of GIT parasites (Table 4). Although not statistically significant, chickens under backyard production system, local breed, and having male sex were relatively more frequently infected with helminth parasites when compared with their relatives.

Results in (Table 5) showed that seven (N = 7) helminth parasite species have been recorded during necropsy examination; three nematodes, namely* Ascaridia galli*,* Heterakis gallinarum, *and Capillaria species, and four cestodes, namely,* Raillietina tetragona, Raillietina echinobothrida, Raillietina cesticillus, *and* Hymenolepis carioca*, were identified.

Among the postmortem examined chickens (N = 138), a prevalence rate of 87.68% (n=121) and 86.96% (n = 120) was recorded for cestodes and nematodes, respectively.  Table 6 attested that the infection rate variation of cestode parasites was statistically significant (p<0.05). The result has elaborated that the infection rate variation of cestode parasites of local breeds under backyard management system (92.7%) was significantly higher than the infection rate variation of cestode parasites of exotic breeds kept under intensive management (73.8%). However, variations among the prevalence of nematode parasites and sex factors of chicken were not statistically significant.

Most of the postmortem examined chicken (87.7%) was harboring adults of two or more species of helminth parasites (mixed infection); the highest burden has been recorded with 5 parasites (21%) in chickens followed by 3 parasites (17%) per a single sample (Table 7). However, obtaining of all identified parasite species in a single chicken (12.32%) is alarming for their economic significance.

As shown in Table 8, similar gastrointestinal helminth parasites infection rate of chicken was obtained from both examination approaches (necropsy, 90.60%, and coproscopy, 90.97%). Although prevalence variation was recorded while diagnosing some parasite species, this may be due to the difference in nature that each adult and/or eggs of the identified parasite species own. This study revealed that* Heterakis gallinarum *(72.5%) and* Ascaridia galli* (68.8%) from nematodes and* Raillietina tetragona* (65.9%) from cestodes were the dominant helminth parasite species identified by necropsy examination.

## 4. Discussion

The present study revealed that 90.60% (125/138) of chickens were positive for one or more helminth parasites during necropsy examination. It is comparable with the findings reported from Barisal district, Bangladesh (91.88%), Nairobi County, Kenya (90%), and Hawassa, Ethiopia (88.5%) [13–15]. However, it was high when compared with reports from Akure, Nigeria (20.5%) [16]; this lower prevalence recorded may be because of the relatively small number of chickens (sample size) examined during the Nigerian study that involved only 85 chickens.

The current study with necropsy examination showed a comparable prevalence of cestode (86.96%) and nematode (87.68%) parasites. However, it is different from findings reported from central Ethiopia (86.32%, 75.79%) and Nairobi County, Kenya (68.1%, 74.4%) for cestode and nematode infections, respectively [9, 17]. The prevalence variations observed among cestodes and nematodes could partly be associated with the chance of chickens to pick intermediate hosts from ground. Besides, there are differences among the countries agroecological and local environment. The absence of trematode parasites during both necropsy and fecal examination of the current study was in agreement with findings reported from Giwa local government, Nigeria and Mbeere subcounty, Kenya [18, 19]. However, trematodes were recorded from Kiambu and Nairobi counties, Kenya [20]. This difference could be due to the absence or lesser occurrence of the snail intermediate hosts responsible for their transmission.

This study showed that vast majority (87.7%) of the chickens in the study area harbored multiple (more than one) species of helminth parasites, in which their combined devastating effects on the host metabolism play a major role in early chick mortality and other production losses among adults [21]. However, this finding was different when comparing with findings reported from both Nsukka region 56% [22] and Giwa local government areas of Nigeria 60.5% [18], which strongly suggests that the prevailing environmental conditions in Ethiopia may be favorable to their simultaneous development. Feeding choice of the chickens may also affect the situation.

The prevalence of* Heterakis gallinarum *(72.5%) and* Ascaridia galli *(68.8%) obtained in the current study was very high when compared with the findings reported from Gharb Region Morocco (10%, 9%), Giwa local government Nigeria (20.5%, 17.0%), and South East Tigray Ethiopia (10%, 35%), respectively [18, 23, 24]. This could explain the higher reproductive potential (fecundity) of female worm. Moreover, the ubiquitous earth worms can ingest eggs of the cecal worms and act as the major means of infection in poultry.

Among the cestode parasite species of chicken revealed from the current study,* Raillietina echinobothrida *was the most abundant one (54.6%) which was lower than that reported from Nsukka region, Nigeria (64.5%) [22]. However, it was much higher than the study conducted in north Gondar zone, Ethiopia, with 29.62 [25] and Iran with 6% prevalence [26]. This variation may be for the difference in agrogeological condition, poultry management system, level of exposure to specific intermediate hosts, and sampling sites. The current study has failed to detect* Davainea proglottina* during necropsy examination; this was in agreement with studies conducted in central Ethiopia [9] and Gaza Strip Palestine [27]. However, a report from Nairobi County Kenya reveals 6.9% prevalence of the parasite [14]. The absence may be due to the small size of the parasite (<4mm) which makes it be missed during examination.

A statistically significant difference was observed in the overall prevalence of gastrointestinal helminths among chickens kept under the backyard, semi-intensive, and intensive management systems. This showed that the prevalence of helminth parasites was decreased with increasing quality and modernization of the production system, which is in agreement with the study conducted in Akure, Nigeria [16]. This might be because of the hygienic environment and feeding system of the chicken in the intensive farms, which is not favorable for helminth parasites growth and transmission [7]. However, in case of the free ranging, chickens move and usually ingest the intermediate hosts in perusing of feed and water. Besides, chickens of the local breeds were also significantly infected when compared with that of exotic ones, which might be because most of the local chickens examined were from that of backyard management system and also variation in chickens feeding behavior. However, there was no statistically significant difference for infection rate among sex, which is in agreement with the studies conducted in Gharb region-Morocco and Giwa local government, Nigeria [18, 23].

## 5. Conclusion

This study had come up with extremely high prevalence of cestode and nematode helminth infections. The majority of chickens were also found harboring more than one helminth parasite species. Management system and breed were identified as major risk factors affecting the occurrence of helminth parasites. These findings will become more detrimental because poultry sector is an infant, but fast growing industry in Ethiopia at large and in Mekelle town in particular, which are highly dominated with local chickens kept under backyard production system. Therefore, urgent intervention with regular deworming scheme and continuous surveillance is critical. Moreover, strict attention should be given towards maintaining hygienic condition and modernizing the management system together with breed improvement measures of chicken.

## Figures and Tables

**Table 1:** Univariate and multivariate regression analysis of helminth parasitism among considered risk factors (coproscopy).

Possible risk factors		Number examined	Number Positive ('%)	Crude OR	P-value	Adjusted OR	P-value
				(CI95%)		(CI95%)	
*Management *	Intensive	125	105 (84.0)	1		1	
	Semi Intensive	130	120 (92.3)	2.28 (1.02, 5.10)	0.04	2.14 (0.85, 5.37)	0.11
	Backyard	155	148 (95.5)	4.02 (1.64,9.87)	0.00	3.40 (0.71,16.31)	0.13
*Breed *	Exotic	213	186 (87.3)	1		1	
	Local	197	187 (94.9)	2.71 (1.28,5.77)	0.01	1.17 (0.31,4.36)	0.82
*Sex *	Female	201	180 (89.6)	1		1	
	Male	209	193 (92.3)	1.41 (0.71,2.78)	0.33	1.06 (0.56,2 17)	0.87

*Overall*		**410**	**373(90.98)**				

**Table 2:** Describing prevalence of helminth parasite Species identified among the considered risk factors (coproscopy).

Possible risk factors		No. examined	A. galli^a^	H. galli^b^	Cap. Spp^c^	R. tetra^d^	R. echino^e^	R. cesti^f^	D. prog^g^	H. cario^h^
*Management*	Intensive	125	62 (49.6)	64 (51.2)	43 (34.4)	69 (55.2)	54 (43.2)	54 (43.2)	57 (45.6)	45 (36.0)
	Semi-intensive	130	93 (71.5)	94 (72.3)	94 (65.4)	50 (38.5)	62 (47.7)	81 (62.3)	85 (63.4)	41 (31.5)
	Backyard	155	118 (76.1)	111 (71.6)	115 (74.2)	112 (72.3)	108 (69.7)	111 (71.6)	124 (80.0)	29 (18.7)
*Breed*	Exotic	213	124 (58.2)	129 (60.6)	102 (47.9)	108 (50.7)	94 (44.1)	107 (50.2)	113 (53.1)	65 (30.5)
	Local	194	149 (75.6)	140 (71.1)	141 (71.6)	123 (62.4)	130 (67.0)	139 (70.6)	153 (77.7)	50 (25.4)
*Sex*	Male	209	150 (71.8)	153 (73.2)	132 (63.2)	118 (56.5)	119 (57.5)	131 (62.7)	137 (65.6)	55 (26.3)
	Female	201	123 (61.2)	116 (57.7)	111 (55.2)	113 (56.2)	105 (52.2)	115 (57.2)	129 (64.2)	60 (29.9)

*Overall parasite species *		**410**	**273(66.6)**	**269(65.6)**	**243(59.3)**	**231(56.3)**	**224(54.6)**	**246(60.0)**	**266(64.9)**	**115(28.1)**

a, *Ascaridia galli*; b, *Heterakis gallinarum; *c, *Capillaria* species; d, *Raillietina tetragona*; e, *Raillietina echinobothrida*; f, *Raillietina cesticillus*; g, *Davania proglottina*; h, *Hymenolepis carioca*. The numbers within the table stand for the following: number positive (prevalence in percentage).

**Table 3:** Univariate and multivariate regression analysis of cestode and nematodes among the considered risk factors (coproscopy).

*Possible Risk factor*		*Number Examined*	*Cestode*			*Nematode*		
			Number	Crud OR	Adjusted OR	Number	Crud OR	Adjusted OR
			Positive (%)	(95% CI)	(95% CI)	Positive (%)	(95% CI )	(95% CI)
*Management*	Intensive	125	100(80.0)	1	1	89(71.20)	1	1
	Semi intensive	130	117(90.0)	2.3(1.1-4.6)	2.3(0.98-5.2)	114(87.7)	2.9(1.5-5.5)	2.6(1.2-5.5)
	Backyard	155	148(95.5)	5.3(2.2-12.7)	4.9(1.1-20.9)	142(91.6)	4.8(2.4-9.8)	4.1(1.2-14.3)
*Breed*	Exotic	213	179(84.0)	1	1	166(77.9)	1	1
	Local	197	186(94.4)	3.2(1.6-6.5)	1.2(0.4-3.9)	179(90.9)	2.9(1.6-5.4)	1.1(0.4-3.1)
*Sex*	Female	201	178(88.6)	1	1	161(80.1)	1	1
	Male	209	187(89.5)	1.1(0.5-1.6)	1.3(0.7-0.5)	184(88.0)	1.8(0.3-0.9)	1.4(0.4-1.3 )

*Overall*		**410**	**365(89.0)**			**346(84.4)**		

**Table 4:** The helminth parasites infection rate in chickens under different factors (necropsy).

Possible Risk Factors		Number Examined.	Number positive	Percentage (%)	P-value
*Management*	Intensive	42	36	85.7	0.20
	Backyard	96	89	92.7	
*Breed*	Exotic	42	36	85.7	0.20
	Local	96	89	92.7	
*Sex*	Male	96	88	91.7	0.79
	Female	42	37	88.1	

*Overall parasitism*		**138**	**125**	**90.6**	

**Table 5:** Describing prevalence of helminth parasite species identified among the different factors considered (necropsy).

Possible Risk factors		Number examined	A. galli^a^	H.galli.^b^	Cap. Spp^c^	R.tetr.^d^	R.echino^e^	R.cesti^f^	H. cario^g^
*Management*	Intensive	42	19 (45.2)	20 (47.6)	16 (38.1)	20 (47.6)	8 (19.1)	14 (33.3)	03 (07.1)
	Backyard	96	76 (79.2)	80 (83.3 )	55 (57.3)	71 (73.9)	47 (48.9)	62 (64.5)	69 (71.9)
*Breed*	Exotic	42	19 (45.2)	20 (47.6)	16 (38.1)	20 (47.6)	08 (19.1)	14 (33.3)	03 (07.1)
	Local	96	76 (79.2)	80 (83.3 )	55 (57.3)	71 (73.9)	47 (48.9)	62 (64.5)	69 (71.9)
*Sex*	Male	96	70 (72.9)	74 (77.1 )	51 (53.1)	63 (65.6)	38 (39.6)	57 (59.4)	56 (58.3)
	Female	42	25 (59.5)	26 (61.9)	20 (47.6)	28 (66.7)	17 (40.5)	19 (45.2)	16 (38.1)

*Overall parasite species*		**138**	**95(68.84)**	**100(72.46)**	**71(51.45)**	**91(65.94)**	**55(39.86)**	**76(55.07)**	**72(52.17)**

a, *Ascaridia galli*; b, *Heterakis gallinarum*; c, *Capillaria species*; d, *Raillietina tetragona*; e, *Raillietina echinobothrida*; f, *Raillietina cesticillus*; g, *Hymenolepis carioca*. The numbers within the table stand for the following: number positive (prevalence in percentage).

**Table 6:** Prevalence of cestode and nematode parasites of chicken among different factors considered (necropsy).

*Possible Risk factor*		*Number Examined*	*Cestode*			*Nematode*		
			Number Positive	Percentage (%)	P-value	Number Positive	Percentage (%)	P- value
*Management*	Intensive	42	31	73.8	0.002	34	80.9	0.112
	Backyard	96	89	92.7		87	90.6	
*Breed*	Exotic	42	31	73.8	0.002	34	80.9	0.112
	Local	96	89	92.7		87	90.6	
*Sex*	Male	96	83	86.4	0.793	85	88.5	0.642
	Female	42	37	88.0		36	85.7	

* Overall*		**138**	**121**	**87.68**		**120**	**86.96**	

**Table 7:** Describing the burden of helminth parasitic infections recorded per chicken (necropsy).

*No*.	*Categories* (No. parasites per sample)	*Frequency* (number)	Percentage (%)
*1*	No infection (0)	13/138	09.42
*2*	Single infection (1)	04/138	02.90
*3*	Two parasites (2)	12/138	08.70
*4*	Three parasites (3)	23/138	16.67
*5*	Four parasites (4)	21/138	15.22
*6*	Five parasites (5)	29/138	21.01
*7*	Six parasites (6)	19/138	13.77
*8*	Seven parasites (7)	17/138	12.32

*Overall Multiple infection (≥2 parasites)*		**121/138**	**87.68**

**Table 8:** Describing prevalence of the identified parasite species using both techniques employed.

*No*	*Helminth species *	* Techniques employed*		* P-value*
		Necropsy (n=138)	Coproscopy (n=410)	
1	*Ascaridia galli*	68.8	0.63	66.6
2	*Heterakis gallinarum*	72.5	65.6	0.13
3	*Capillaria species*	51.5	59.3	0.11
4	*Raillietina tetragona*	65.9	56.3	0.04
5	*Raillietina echinobothrida*	39.9	54.6	0.00
6	*Raillietina cesticillus*	55.1	60.0	0.31
7	*Hymenolepis carioca*	52.2	28.1	0.00

	*Overall prevalence*	**90.60**	**90.98**	

## Data Availability

The raw data used to support the findings of this study have been deposited in the Mendeley data repository (doi: 10.17632/dyk9b8wpcx.1). The available data can be cited as Gebremedhin, Mebrahtu Berhe (2018), “Helminth parasites of chicken (raw data)”, Mendeley Data, v1 https://dx.doi.org/10.17632/dyk9b8wpcx.1.
